# Large‐scale network dysfunction in vascular cognitive disorder supports connectional diaschisis in advanced arteriosclerosis

**DOI:** 10.1111/ene.14084

**Published:** 2019-10-25

**Authors:** D. Meng, A. A. Hosseini, R. J. Simpson, T. Welton, R. A. Dineen, D. P. Auer

**Affiliations:** ^1^ Radiological Sciences Division of Clinical Neuroscience School of Medicine Queen's Medical Centre University of Nottingham Nottingham UK; ^2^ Sir Peter Mansfield Imaging Centre School of Medicine University of Nottingham Nottingham UK; ^3^ NIHR Nottingham Biomedical Research Centre Queen's Medical Centre University of Nottingham Nottingham UK; ^4^ Department of Vascular Surgery Queen's Medical Centre Nottingham University Hospitals NHS Trust Nottingham UK

**Keywords:** cohort study, functional magnetic resonance imaging, vascular dementia

## Abstract

**Background and purpose:**

The interrelation of cognitive performance, cerebrovascular damage and brain functional connectivity (FC) in advanced arteriosclerosis remains unclear. Our aim was to investigate the associations between FC, white matter damage and cognitive impairment in carotid artery disease.

**Methods:**

Seventy‐one participants with a recent cerebrovascular event and with written informed consent underwent resting‐state functional magnetic resonance imaging and the Addenbrooke's Cognitive Examination – Revised (ACE‐R). Network and inter‐hemispheric FC metrics were compared between cognitively normal and impaired subjects, and interrelated with cognition. In order to explore the nature of FC changes, their associations with microstructural damage of related white matter tracts and cognitive performance were investigated, followed by mediation analysis.

**Results:**

Participants with global cognitive impairment showed reduced FC compared to the cognitively intact subjects within the central executive network (CEN), and between hemispheres. Patients with executive dysfunction had decreased CEN FC whilst patients with memory loss demonstrated low FC in both the CEN and the default mode network (DMN). Global performance correlated with connectivity metrics of the CEN hub with DMN nodes, and between hemispheres. Cingulum mean diffusivity (MD) was negatively correlated with ACE‐R and CEN–DMN FC. The cingulum MD–cognition association was partially mediated by CEN–DMN FC.

**Conclusions:**

Long‐range functional disconnection of the CEN with DMN nodes is the main feature of cognitive impairment in elderly subjects with symptomatic carotid artery disease. Our findings provide further support for the connectional diaschisis concept of vascular cognitive disorder, and highlight a mediation role of functional disconnection to explain associations between microstructural white matter tract damage and cognitive impairment.

## Introduction

Vascular cognitive disorder (VCD) is common in people 65 years of age and older (prevalence from 2.2% to 16.3%) 1 and encompasses a range of cognitive disorders that have a presumed vascular cause which may be small vessel disease, territorial infarctions or haemorrhage. The pathophysiology of VCD is not fully understood but radiological evidence supports disconnection of cognitive networks as a key mechanism that may result from focal (‘strategic’) vascular injury of the involved network hubs or from damage of interconnecting white matter pathways 2, 3. Recent reports on disruptions of multiple brain networks and inter‐hemispheric functional connectivity (FC) in VCD suggest direct associations between FC changes and cognitive impairment 4, 5, 6, 7, 8, 9 thereby supporting the notion that structural or functional disconnection of areas distant from the lesion may be one of the main causes of VCD, which has been framed as ‘connectional diaschisis’ 10. However, the hallmarks of brain network dysfunctions that underpin VCD in advanced arteriosclerosis and their interrelation with structural tissue damage remain under‐researched.

It is hypothesized that, regardless of the vascular subtype in people with VCD, distinct patterns of network disruption underpin cognitive dysfunction and are associated with brain tissue damage. The functional architecture of the brains of people with symptomatic carotid artery disease with and without cognitive impairment was characterized and compared using established network metrics. Using a mediation analysis, the nature of the interrelation between functional disconnection, mean diffusivity (MD) of white matter tracts (WMTs) and cognition was also investigated.

## Methods

### Study population

The patients were a subgroup of a previously reported cohort 3, 11, 12. Participants with a recent non‐disabling cerebrovascular event (stroke, transient ischaemic event or amaurosis fugax), ipsilateral carotid stenosis of >30% and not eligible for carotid endarterectomy underwent cognitive and magnetic resonance imaging (MRI) assessment. Patients with ≥80% stenosis and either clinical or radiological (watershed infarcts) signs of haemodynamic impairment were excluded. The study was approved by the local Research Ethics Committee and all participants gave written informed consent.

### Image protocol

Subjects underwent MRI at 3 T using a 16‐channel neurovascular coil (Achieva, Philips Medical Systems, Amsterdam, The Netherlands) with the protocol including eye‐closed resting‐state functional MRI (fMRI), axial diffusion tensor imaging (DTI) and axial fluid‐attenuated inversion recovery (FLAIR). Protocol parameters are included in Appendix S1.

To account for possible artefacts resulting from micro‐motion, a rigorous protocol of data quality assessment 13 was applied, as detailed in Appendix S1, resulting in a final dataset of 71 subjects.

### Cognitive assessment

All participants underwent cognitive assessment on the day of enrolment using Addenbrooke's Cognitive Examination – Revised (ACE‐R) by trained research investigators and patients with probable VCD were classified as previously described 3. Participants with executive dysfunction or memory impairment were defined based on the ACE‐R subscales (Appendix S2).

### Large‐scale and short‐range FC analyses

Functional MRI data were subjected to standard pre‐processing (slice timing correction, realignment, motion correction, normalization and smoothing) using the FMRIB Software Library (FSL, Oxford, UK) 14 and then identifying resting‐state networks (RSNs) using multi‐session temporal concatenation probabilistic independent component analysis (ICA) 15. All independent components were visually compared with published RSNs for further analyses 16. Individual regional *Z* scores were extracted from each individual's *Z*‐statistic map. Five RSNs considered to be related to cognition 17 were selected for further analyses: the frontoparietal network consisting of the intraparietal sulcus, inferior parietal lobe and dorsal premotor cortex, involved in category‐based, object‐based, feature‐based and space‐based attention selection; the default mode network (DMN) formed by the posterior cingulate cortex (PCC)/precuneus, medial prefrontal cortex, angular gyrus, lateral and anterior temporal cortex and hippocampus; the central executive network (CEN) composed of the dorsolateral prefrontal cortex and posterior parietal cortex; the salience network including the dorsal anterior cingulate cortex; and the insula networks, considered to be central for higher cognitive functions.

Homologous and heterologous inter‐hemispheric FCs of the RSNs were investigated using a standard approach by averaging FC values of homologous and heterologous regions of interest within RSNs, as detailed in Appendix S3.

### Anatomical markers of WMT damage

Mean diffusivity was chosen as a reliable and sensitive measure of WMT injury after stroke 18. WMTs underlying or connected to the significantly altered FC clusters were identified by overlapping the white matter tractography atlas in FSL 14 onto the cluster maps showing significant large‐scale RSN abnormalities.

### Total ischaemic lesion load and temporal lobe atrophy

Total ischaemic lesion load was defined as a conjunction of acute, chronic ischaemic and infarcted lesion volumes, normalized for total intracranial volume brain, as previously reported for this patient cohort 3, 11, 12. In brief, individual lesion maps were created by manually outlining acute (diffusion weighted imaging positive lesions) and chronic ischaemic (hyperintense on FLAIR) and infarcted (hypointense on FLAIR) lesions. Total intracranial volume was measured with a package in FSL 19.

Medial temporal atrophy was assessed on reconstructed coronal FLAIR images using Schelten's scale; results for this patient group were previously reported 3, 11.

### Statistical analysis

The independent‐samples *t* test, Mann–Whitney *U* test and chi‐squared test in SPSS 21(IBM, NY, USA) were used to compare the clinical characteristics.

All statistical tests of FC metrics were controlled for age and mean relative displacement. Standard dual regression analysis in FSL 20 was used to compare large‐scale network characteristics between cognitively impaired and intact subjects. Inference for voxel‐based ICA was based on general linear models with permutation tests (*n* = 5000). Significance was considered at *P* < 0.05 corrected using the family‐wise error rate.

SPSS 21 was used for (i) *post hoc* correlation analysis between RSN‐specific individual FC (indexed as *Z* score) in regions showing differences between cognitive subgroups and ACE‐R scores; (ii) *post hoc* between‐group comparison and cognitive correlation of homologous and heterologous inter‐hemispheric FC; (iii) *post hoc* correlation analysis between ultrastructural damage of selected WMTs and functional disconnection. The Benjamini–Hochberg procedure was applied to correct for the false discovery rate (FDR) 21 in all *post hoc* correlation analyses.

The PROCESS macro ( http://processmacro.org/index.html) for SPSS 21 was used to conduct the mediation analysis to investigate whether functional disconnection of RSNs mediates the effect of ultrastructural damage of WMTs on cognition. The significance of indirect effects was tested using bootstrapping with 1000 replications. The percentage of the mediator effect accounting for the total effect was calculated and reported.

## Results

Seventy‐one subjects [age, mean ± SD, 74.2 ± 10.2; female 26 (36.6%), 40 cognitively intact and 31 (43.7%) probable VCD] were included. Patients with probable VCD were more likely to have a history of smoking (*P* = 0.003) and tended to be older (*P* = 0.05) (Table 1).

**Table 1:** Comparison of clinical characteristics between patients with normal and abnormal cognition

Characteristics	Normal (*n* = 40)	Abnormal (*n* = 31)	*P*
Age, mean (SD) (years)	72.1 (10.6)	76.7 (9.1)	0.05
Female, *n* (%)	12 (30.0)	14 (45.2)	0.22
Atrial fibrillation, *n* (%)	8 (20.0)	8 (25.8)	0.58
Peripheral vascular disease, *n* (%)	4 (10.0)	8 (25.8)	0.11
Ischaemic heart disease, *n* (%)	10 (25.0)	11 (35.5)	0.43
Hypertension, *n* (%)	30 (75.0)	26 (83.9)	0.39
Smoking, *n* (%)	19 (47.5)	26 (83.9)	0.003^a^
Diabetes mellitus, *n* (%)	8 (20.0)	9 (29.0)	0.41
Symptomatic type (stroke), *n* (%)	20 (50.0)	16 (51.6)	0.89
Symptomatic side (left), *n* (%)	18 (45.0)	12 (38.7)	0.63
Ipsilateral degree of carotid stenosis, *n* (%)			
30%–49%	20 (50.0)	17 (54.8)	0.92
50%–59%	9 (22.5)	5 (16.1)	
60%–69%	4 (10.0)	5 (16.1)	
70%–79%	3 (7.5)	2 (6.5)	
80%–89%	1 (2.5)	1 (3.2)	
>90%	3 (7.5)	1 (3.2)	
Contralateral degree of carotid stenosis, *n* (%)			
0%–29%	30 (75.0)	21 (67.7)	0.26
30%–49%	4 (10.0)	1 (3.2)	
50%–59%	4 (10.0)	5 (16.1)	
60%–69%	2 (5.0)	2 (6.5)	
70%–79%	0 (0)	1 (3.2)	
80%–89%	0 (0)	0 (0)	
>90%	0 (0)	3 (9.7)	
Time from cerebrovascular ischaemic event to MRI, mean (SD) (days)	35.2 (32.4)	40.4 (41.3)	0.56
Medial temporal lobe atrophy	6 (15.0)	12 (38.7)	0.06
Log TILL^b^	−5.3 (1.5)	−5.1 (1.4)	0.63
Percentage normalized volume of lacunar infarction, ×10^−5^ (SD)	1.0 (2.0)	0.8 (2.0)	0.722
Percentage normalized volume of DWI hyperintense lesions, ×10^−4^ (SD)	3.7 (7.8)	5.6 (13.2)	0.475
Percentage normalized volume of white matter hyperintense lesions, ×10^−3^ (SD)	11.02 (12.71)	12.54 (15.09)	0.653

DWI, diffusion weighted imaging.

^a^Significant level at *P* < 0.05.

^b^log TILL, logarithmic total ischaemic lesion load (DWI hyperintense, FLAIR hyperintense and hypointense lesions) normalized to intracranial volume.

### Functional disconnection of large‐scale RSNs in probable VCD

**Figure 1:**
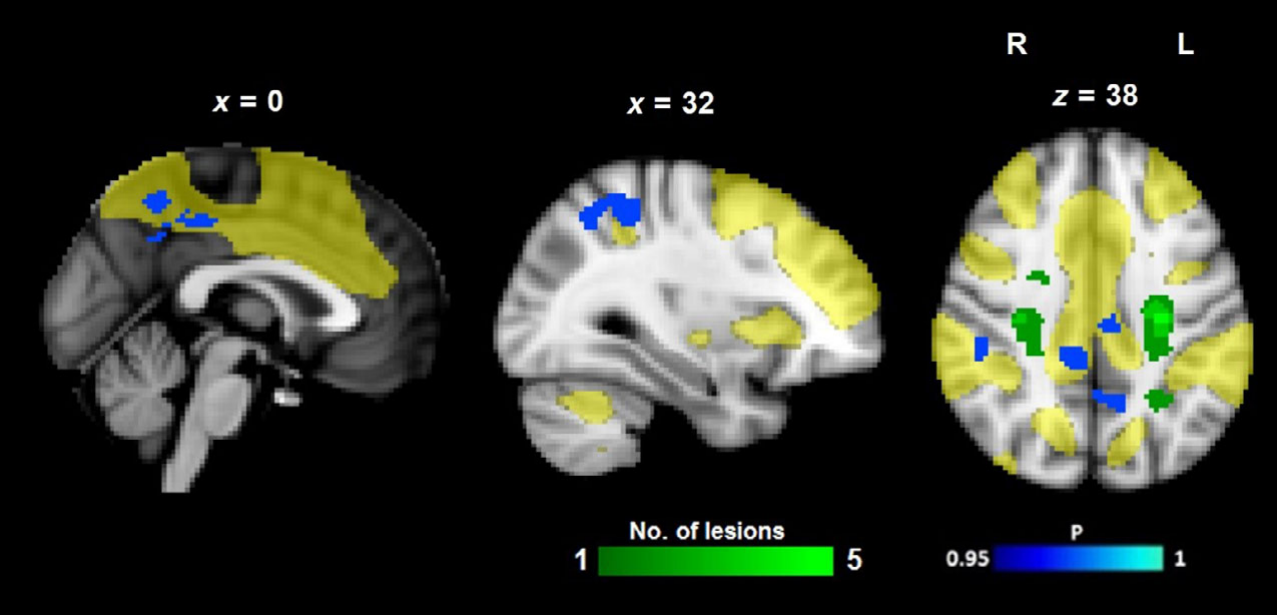
Resting‐state network maps and global cognition in patients with symptomatic carotid artery disease. A difference map (blue–light blue) of the CEN (yellow mask overlaid onto the 2 × 2 × 2 mm MNI 152 template) where patients with probable vascular cognitive disorder had decreased functional connectivity compared with patients with normal cognition. The blue–light blue colour bar shows the range of family‐wise error‐corrected *P* value. The green–light green colour bar shows the variation between the minimum and maximum number of acute lesions on DTI b0 images. All tests were corrected for age and mean relative displacement.

A set of 17 independent components was obtained using probabilistic ICA with temporal concatenation 16. Patients with probable VCD had reduced CEN FC in the PCC, precuneus and right parietal lobule (Fig. 1, Table 2), compared to cognitively intact patients.

**Table 2:** Brain regions showing decreased functional connectivity within resting‐state networks in patients with probable vascular cognitive disorder versus cognitively intact reference group

	Coordinates (mm)			*P* value	Correlation with cognitive performance	
	*X*	*Y*	*Z*		*r*	*P* a
Global cognition						
Precuneus	−12	−56	38	0.028	0.367	0.002
PCC	4	−38	40	0.034	0.369	0.002
Right parietal lobule	32	−60	46	0.037	0.251	0.037

PCC, posterior cingulate cortex.

a False discovery rate corrected *P* = 0.05.

*Post hoc* analysis of FC limited to those areas showing less CEN FC in the probable VCD group revealed multiple significant correlations between functional disconnection and global cognitive impairment but no significant correlations between functional disconnection and white matter hyperintensity volume (*P* = 0.934; Table 2, Fig. S2 A–C).

Details of functional disconnection of large‐scale RSNs in subgroups defined by deficits in two relevant cognitive subdomains (executive function and memory) are provided in Appendix S4.

### Reduced inter‐hemispheric FC in probable VCD

**Figure 2:**
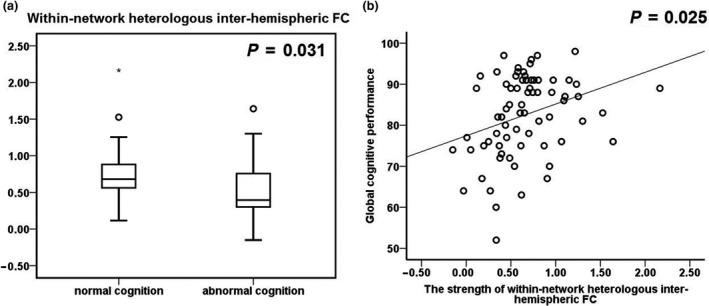
Inter‐hemispheric FC and cognition in patients with symptomatic carotid artery disease. (a) Box plots showing network‐averaged heterologous inter‐hemispheric FC in patients with probable VCD and with normal cognition. (b) Scatterplots showing correlation between ACE‐R scores and network‐averaged heterologous inter‐hemispheric FC, adjusted for age.

Within‐network heterologous inter‐hemispheric FC was significantly decreased in probable VCD patients compared to cognitively intact patients (partial *η* ^2^ = 0.067, *P* = 0.031, Fig. 2). The strength of heterologous (*r* = 0.270, *P* = 0.025) inter‐hemispheric FC was significantly and positively correlated with ACE‐R (Fig. 2).

### The role of WMT damage in functional network disconnection

Fifty‐one subjects (71.8%) had DTI scans with good quality 3 thus allowing the structural integrity of WMTs to be investigated, and indexed as WMT MD.

Based on the approach of selecting WMTs underlying functional disconnection, the cingulum and right superior longitudinal fasciculus (SLF) were identified for the CEN (Fig. S3). The corpus callosum (CC) MD was investigated for associations with inter‐hemispheric homologous and heterologous RSN FC.

Addenbrooke's Cognitive Examination – Revised was significantly and negatively related to cingulum MD (*r* = −0.301, *P* = 0.001) but not right SLF (*P* = 0.295). Also, cingulum MD and FC were significantly anti‐correlated in CEN PCC (*r* = −0.326, *P* = 0.018) and CEN precuneus [*r* = −0.377, *P* = 0.006 (FDR‐corrected *P* = 0.03)]. No significant correlation between right SLF MD and right parietal lobule FC was identified. CC MD was correlated with heterologous (*r* = −0.301, *P* = 0.028) but not homologous (*P* = 0.303) inter‐hemispheric FC.

### Mediation effects of FC on the association between structural integrity and cognition

**Figure 3:**
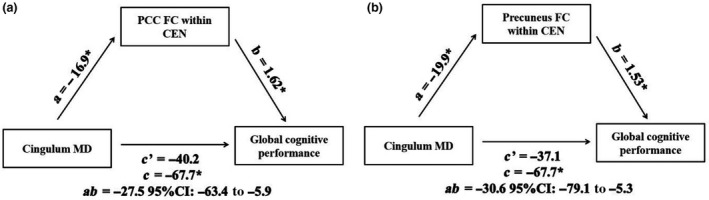
Mediation model of associations between (a) PCC FC within the CEN, cingulum MD and global cognitive performance; (b) precuneus FC within the CEN, cingulum MD and global cognitive performance. The values (*a, b, c, c′*) presented are unstandardized regression coefficients. Path *c* represents the total effect. Path *c′* represents the direct effect. *ab* is the indirect effect. *Significant correlation at *P* < 0.05.

Mediation analysis was conducted to investigate the association between brain networks’ FC, WMT integrity and ACE‐R. Regional FC within RSNs or between hemispheres that showed decreased FC in probable VCD was selected as possible mediators. PCC FC within the CEN accounted for 40.6% of the total effect of cingulum MD on ACE‐R, and precuneus FC within the CEN accounted for 45.2% of the total effect of cingulum MD on ACE‐R (Fig. 3). The FC of PCC or precuneus with the CEN hub seed did not mediate the association between SLF MD and ACE‐R. Within‐network inter‐hemispheric FC did not mediate the association between CC MD and ACE‐R.

## Discussion

Long‐range FC alterations were characterized in relation to cognition and microstructural WMT injuries in patients with symptomatic carotid artery disease. Functional disconnection of the CEN including its inter‐hemispheric desynchronization was the main abnormality in support of long‐range connectivity diaschisis in VCD. It was also found that functional CEN disconnection was associated with cingulum MD, and partially mediated the link between global cognition and cingulum MD. Moreover, the pattern of functional disconnection varied with the affected cognitive domains.

Central executive network functional disconnection was demonstrated in patients with carotid artery disease with global cognitive impairment in keeping with CEN's known involvement in working memory and cognitive control of thought, emotion and behaviour 22. The role of functional disconnection of the CEN in VCD was further supported by an anti‐correlation between network FC and cognitive performance. Moreover, different CEN FC disconnection patterns were reported for the executive dysfunction and amnestic VCD subtypes suggesting cognitive endophenotypic associations.

Additionally, DMN functional disconnection was found in the amnestic subgroup compared to those with intact memory. Functional disconnection of the DMN has been found in Alzheimer's disease (AD) and mild cognitive impairment 23, 24. The consistent findings of DMN functional disconnection in AD, mild cognitive impairment and amnestic VCD further highlight a specific link between functional DMN disruption and memory loss, pointing to domain rather than disease‐specific disconnections.

The second objective of our study was to characterize the interrelations between structural disconnection, brain network functional disconnection and global cognitive impairment. A direct association between structural and functional disconnection was shown for lacunar infarctions in cerebral autosomal dominant arteriopathy with subcortical infarcts and leukoencephalopathy (CADASIL) 25. In our study, an association of network disruption with tract‐specific microstructural damage was found, indexed as MD increase. To explore the nature of these interrelations between microstructural and functional disconnection and cognition, it is of interest to investigate to what degree any one imaging parameter may mediate the cognitive effects of the other. It was hypothesized that WMT damage is the primary event resulting from microscopic vascular damage, and that this may result in desynchronization in linked long‐range brain networks being impaired. This is likely to lead to failure to efficiently synthesize information from and integrate within several functional brain networks each processing information across anatomically distant brain regions, which in turn provides a plausible mechanism for impaired brain function manifesting as cognitive impairment. This is an important step for a better pathophysiological understanding and selection of imaging biomarkers to predict and assess future clinical trials. Using mediation analysis, it was shown that decreased FC may partially explain the association between microstructural damage of the cingulum and global cognitive impairment. Whilst mediation analysis does not allow inference on causality, given the pathophysiological context of VCD, our findings suggest that reduced FC may be an intermediary mechanism by which structural damage exerts its effect on cognition. This concords with a reported mediation effect of the frontoparietal network FC on the association between lacunar volume and executive dysfunction 25. Therefore, it is proposed that microstructural WMT damage may lead to global cognitive impairment in VCD via impaired synchronization in long‐range brain networks, regardless of the underlying vascular aetiology.

Heterologous inter‐hemispheric FC was reduced in VCD and correlated with the degree of global cognitive impairment, which provides further evidence that inter‐hemispheric neural communications based on preferentially symmetric inhibitory or excitatory control may be critical for cognition. Inter‐hemispheric desynchronization in probable VCD in the subacute phase was shown, demonstrating that the previously observed association of reduced inter‐hemispheric FC with cognitive impairment 26 may persist beyond the acute phase and expected trans‐hemispheric diaschisis. Interestingly, it was found that only heterologous inter‐hemispheric FC was associated with cognition. This might be explained by different dynamic network properties between homologous and heterologous FC. In fact, a recent study reported that homologous FC has lower temporal characteristics (i.e. variability) than heterologous FC 27.

The main limitation of this study is its cross‐sectional observational nature that does not allow inference on causal relationships. Secondly, premorbid neurodegenerative pathology cannot be completely ruled out due to the lack of direct diagnostic tools for amyloid pathology. In fact, the prevalence of coexisting AD and vascular pathology is expected to be 11%–20% in those aged 65 and older 28 reflecting the mixed pathologies that may underlie a subgroup of patients clinically diagnosed as probable VCD.

It is shown that long‐range functional disconnection within the CEN and between hemispheres is linked to global cognitive impairment in probable VCD in patients with symptomatic carotid artery disease. Lastly, it is reported that functional disconnection has a partial mediation effect on the link between microstructural cingulum damage and global cognitive impairment, which suggests that functional neuromodulatory interventions 29 could have therapeutic potential in VCD.

## Disclosure of conflicts of interest

The authors declare no financial or other conflicts of interest.

## Supporting information


**Table S1.** Anatomical labels of each region of interest shown in Fig. S1
**Table S2.** Brain regions showing decreased functional connectivity within resting‐state networks in patients with normal executive function versus executive dysfunction and normal memory versus abnormal memory
**Figure S1.** Regions of interest of RSNs for inter‐hemispheric FC analysis
**Figure S2.** Scatterplots showing the correlation between the strengths of FC within areas showing abnormal FC in the VCD group and cognitive performance.
**Figure S3.** White matter tracts (WMTs) that underlie or connect with identified hubs of CEN were overlaid onto the MNI template.
**Figure S4.** Resting‐state network (RSN) changes related to deficits in cognitive domains in patients with symptomatic carotid artery disease.
