# Supplementary material for: Large‐scale network dysfunction in vascular cognitive disorder supports connectional diaschisis in advanced arteriosclerosis
Source: Eur J Neurol. 2019 Oct 25;27(2):352–9. doi: 10.1111/ene.14084 (PMC6973074; doi:10.1111/ene.14084)
Supplement: Supplementary file 1 — Table S1. Anatomical labels of each region of interest shown in Fig. S1 Table S2. Brain regions showing decreased functional connectivity within resting‐state networks in patients with normal executive function versus executive dysfunction and normal memory versus abnormal memory Figure S1. Regions of interest of RSNs for inter‐hemispheric FC analysis Figure S2. Scatterplots showing the correlation between the strengths of FC within areas showing abnormal FC in the VCD group and cognitive performance. Figure S3. White matter tracts (WMTs) that underlie or connect with identified hubs of CEN were overlaid onto the MNI template. Figure S4. Resting‐state network (RSN) changes related to deficits in cognitive domains in patients with symptomatic carotid artery disease. [file ENE-27-352-s001.docx]

## **Electronic supplementary material**

**Appendix S1**

The MRI protocol included resting-state fMRI (eye-closed, TR/TE 2200ms/35ms, FOV: 208×105×208 mm, 54 slices of 3mm thickness, 150 volumes), axial DTI (spin echo single-shot diffusion-weighted EPI, b [gradient factor] = 1000 s/mm2, 1 b0 volume, 15 directions, TR/TE 4.2 s/57ms, FOV 224 × 224, matrix 112 × 112, 45 slices of 3mm thickness) and axial FLAIR (TR/TE/TI: 10.5/0.14/2.75s, FOV 224 × 192, matrix 224 × 181, 45 slices of 3mm thickness).

Seven participants’ raw resting-state fMRI data were rejected due to artefacts demonstrated by the quantitative output of this QA pipeline and visual inspection of the power spectrum. Pre-processing of resting-state fMRI datasets that had passed QA was performed using MELODIC (multivariate exploratory linear optimized decomposition into independent components) which included motion correction, slice timing correction, brain extraction, spatial smoothing and high-pass temporal filtering. A further ten subjects’ resting-state fMRI datasets were excluded due to excessive mean motion (>3.0mm in mean total displacement or 2.5 degrees in mean total rotation). This resulted in a final dataset of seventy-one subjects that were subjected to standard noise reduction procedures using ICA in MELODIC. This step individually decomposed the fMRI dataset into independent spatio-temporal components that were visually inspected to classify components into those reflecting neural networks and those reflecting physiological noise or movement artefacts, the latter being manually removed.

**Appendix S2**

Patients with executive dysfunction were defined as having impaired function either in fluency(≤9/14 in those aged 50-59 years, ≤8/14 in 60-69 years, ≤9/14 in those over 70 years) or attention(≤17/18 in those aged 50-69 years, ≤16/18 in those over 70 years) using the ACE-R subscales. The executive function score was calculated by the mean of fluency and attention subscales. Patients with memory impairment were classified using the ACE-R memory subscales (≤18/26 in those aged 50-59 years; ≤19/26 in 60-69 years; ≤17/26 in those over 70 years (1, 2)).

**Appendix S3**

Circular regions of interest (ROIs) of fixed size (216mm^3^ [27voxels] were centred on the core regions with the highest Z scores at the group level of each hub of RSNs obtained using the ICA approach (Supplementary Table 1 and Figure 1). The averaged time series of each ROI were extracted and the FC strength of paired ROIs was defined as the correlation coefficient between them, calculated by an in-house matlab script. Nuisance regressors including white matter, CSF and six motion parameters were regressed out. Homologous inter-hemispheric FC was defined as the average of FC strength (indexed as Z scores) between homologous regions in the left and right hemispheres. Heterologous inter-hemispheric FC was the average of FC strength between each ROI and all other ROIs in the same network in the opposite hemisphere, excluding the homologous ROI. Individual Z scores showing the strength of homologous and heterologous inter-hemispheric FC were extracted for each RSN. Homologous and heterologous inter-hemispheric FC at the whole brain level was calculated by averaging the homologous and heterologous inter-hemispheric FC of each RSN.

**Appendix S4**

Patients with executive dysfunction had less FC in the CEN as compared to patients with normal executive function, mainly locating in the bilateral occipital lobe, right parahippocampal gyrus and right caudate (Supplementary Figure 4; Supplementary Table 2). No significant correlation was identified between FC limited to these areas and scores of executive function.

Compared with patients with normal memory, those with impaired memory had decreased FC in the CEN and DMN. Decreased FC within the CEN mainly located in the PCC, ACC, left middle frontal gyrus, left precentral gyrus, bilateral superior frontal gyrus and right occipital lobe. FC of PCC and right occipital lobe within the DMN was decreased in patients with impaired memory. Post-hoc analysis revealed significant correlation between FC of PCC (r=0.416, P<0.001[FDR-corrected P=0.011]) and right occipital lobe and memory performance (r=0.393, P=0.001) (Supplementary Figure 4; Supplementary Table 2).

## **Table S1** Anatomical labels of each ROI shown in Supplementary Figure 1

|  | Anatomical labels | Paired ROIs for homologous inter-hemispheric FC^a^ | Paired ROIs for heterologous inter-hemispheric FC^b^ |
| --- | --- | --- | --- |
| DMN | 1. Left lateral parietal region  2. Left medial prefrontal cortex  3. Left posterior cingulate cortex (PCC)  4. Right lateral parietal region  5. Right medial prefrontal cortex  6. Right PCC | ROI1-ROI4  ROI2-ROI5  ROI3-ROI6 | ROI1-ROI5  ROI1-ROI6  ROI2-ROI4  ROI2-ROI6  ROI3-ROI4  ROI3-ROI5 |
| CEN | 1. Left dorsal lateral prefrontal cortex (DLPFC)  2. Left posterior parietal cortex  3. Right DLPFC  4. Right posterior parietal cortex | ROI1-ROI3  ROI2-ROI4 | ROI1-ROI4  ROI2-ROI3 |
| SAL | 1. Left anterior cingulate cortex (ACC)  2. Left insular  3. Right ACC  4. Right insular | ROI1-ROI3  ROI2-ROI4 | ROI1-ROI4  ROI2-ROI3 |
| Left FPN | 1. Left inferior parietal region  2. Left middle frontal gyrus (MFG)  3. Left PCC  4. Left superior frontal gyrus (SFG)  5. Right inferior parietal region | ROI1-ROI5 | ROI2-ROI5  ROI3-ROI5  ROI4-ROI5 |
| Right FPN | 1. Right inferior parietal region  2. Right MFG  3. Right PCC  4. Right SFG  5. Left inferior parietal region | ROI1-ROI5 | ROI2-ROI5  ROI3-ROI5  ROI4-ROI5 |

a.The homologous interhemispheric FC=sum of paired ROIs FC/the number of paired ROIs. Paired ROIs FC was indexed as Z-transformed correlation coefficient of paired ROIs.

b.The heterologous interhemispheric FC=sum of paired ROIs FC/the number of paired ROIs. Paired ROIs FC was indexed as Z-transformed correlation coefficient of paired ROIs.

## **Table S2** Brain regions showing decreased functional connectivity within resting-state networks in patients with normal executive function vs. executive dysfunction and normal memory vs. abnormal memory

|  |  | | **Coordinates (mm)** | | | **P value** |
| --- | --- | --- | --- | --- | --- | --- |
|  |  |  | X | Y | Z |  |
| Executive function |  |  | |  |  |  |
| Left occipital lobe |  | -14 | | -72 | 22 | 0.029 |
| Right occipital lobe |  | 18 | | -70 | 22 | 0.014 |
| Right parahippocampal gyrus |  | 26 | | -40 | -12 | 0.023 |
| Right caudate |  | 4 | | 12 | -6 | 0.015 |
| Memory-CEN |  |  | |  |  |  |
| Posterior cingulate cortex |  | -2 | | -8 | 32 | 0.012 |
| Anterior cingulate cortex |  | -10 | | 26 | 32 | 0.038 |
| Left middle frontal gyrus |  | -40 | | 36 | 32 | 0.042 |
| Left precentral gyrus |  | -44 | | 2 | 46 | 0.031 |
| Left superior frontal gyrus |  | -12 | | -6 | 64 | 0.016 |
| Right superior frontal gyrus |  | 22 | | -6 | 62 | 0.017 |
| Right occipital lobe |  | 30 | | -66 | -12 | 0.035 |
| Memory-DMN |  |  | |  |  |  |
| Posterior cingulate cortex |  | 6 | | -50 | 38 | 0.045 |
| Right occipital lobe |  | 12 | | -60 | 10 | 0.048 |

**Supplementary Figures:**

**
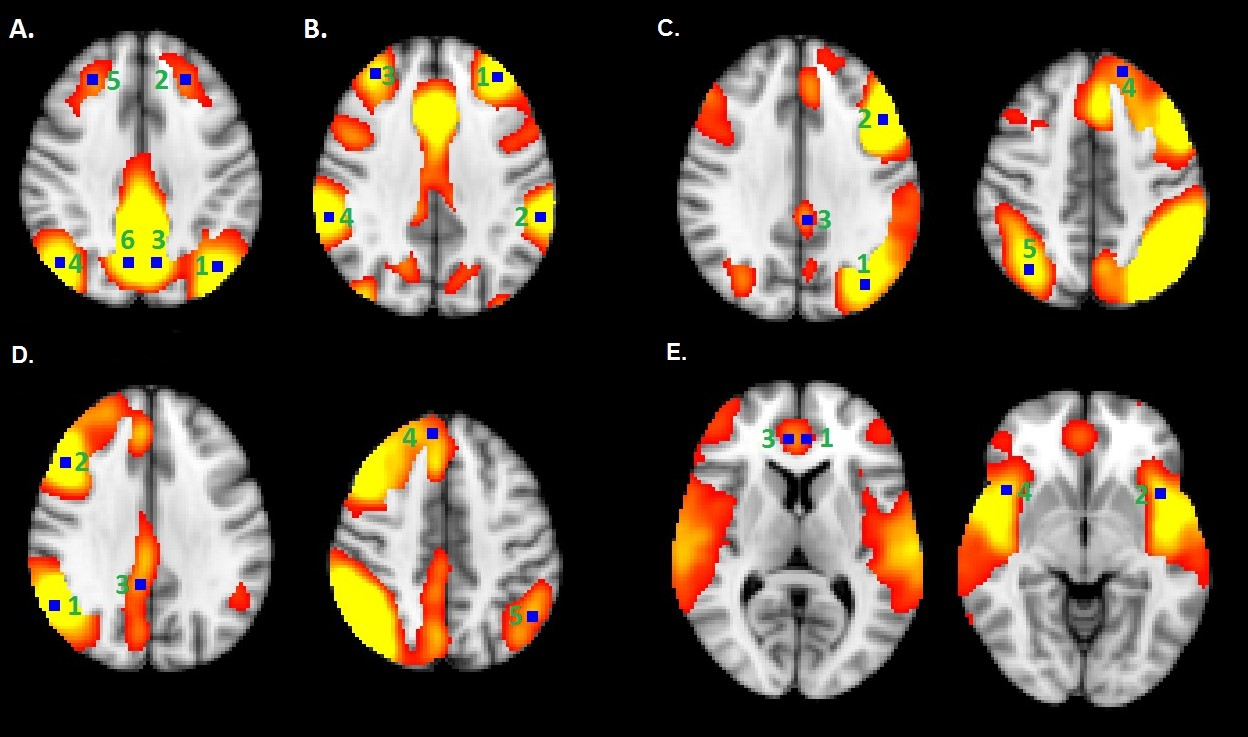
**

**Figure S1** ROIs of RSNs for interhemispheric FC analysis. (A) DMN; (B) CEN; (C) Left FPN; (D) Right FPN; (E) SAL. The anatomical labels of each ROI were provided in Supplementary Table 1.


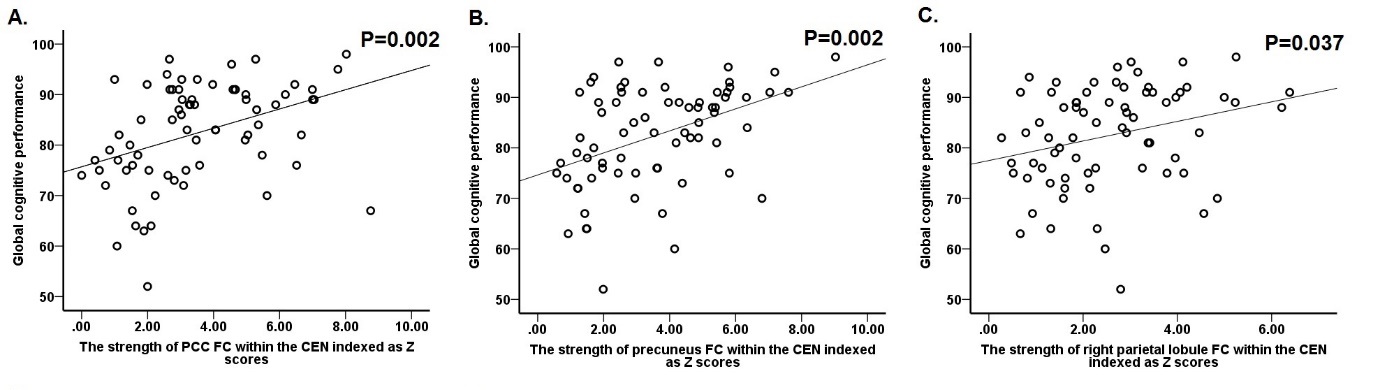


**Figure S2** Scatterplots showing the correlation between the strengths of FC within areas showing abnormal FC in the VCD group and cognitive performance. (A) Scatterplot of the correlation between global cognitive performance and the FC strength of PCC with the CEN. (B) Scatterplot of the correlation between global cognitive performance and the FC strength of precuneus with the CEN. (C) Scatterplot of the correlation between global cognitive performance and FC strength of right parietal lobule with the CEN.


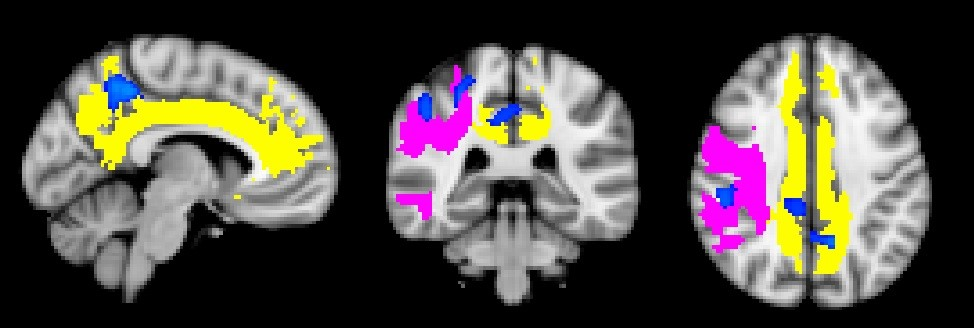


**Figure S3** White matter tracts (WMTs) that underlie or connect with identified hubs of CEN were overlaid onto MNI template. Clusters of decreased FC within CEN in probable VCD patients (Blue) overlapped with or connected to the cingulum (yellow) and right superior longitudinal fasciculus (pink).


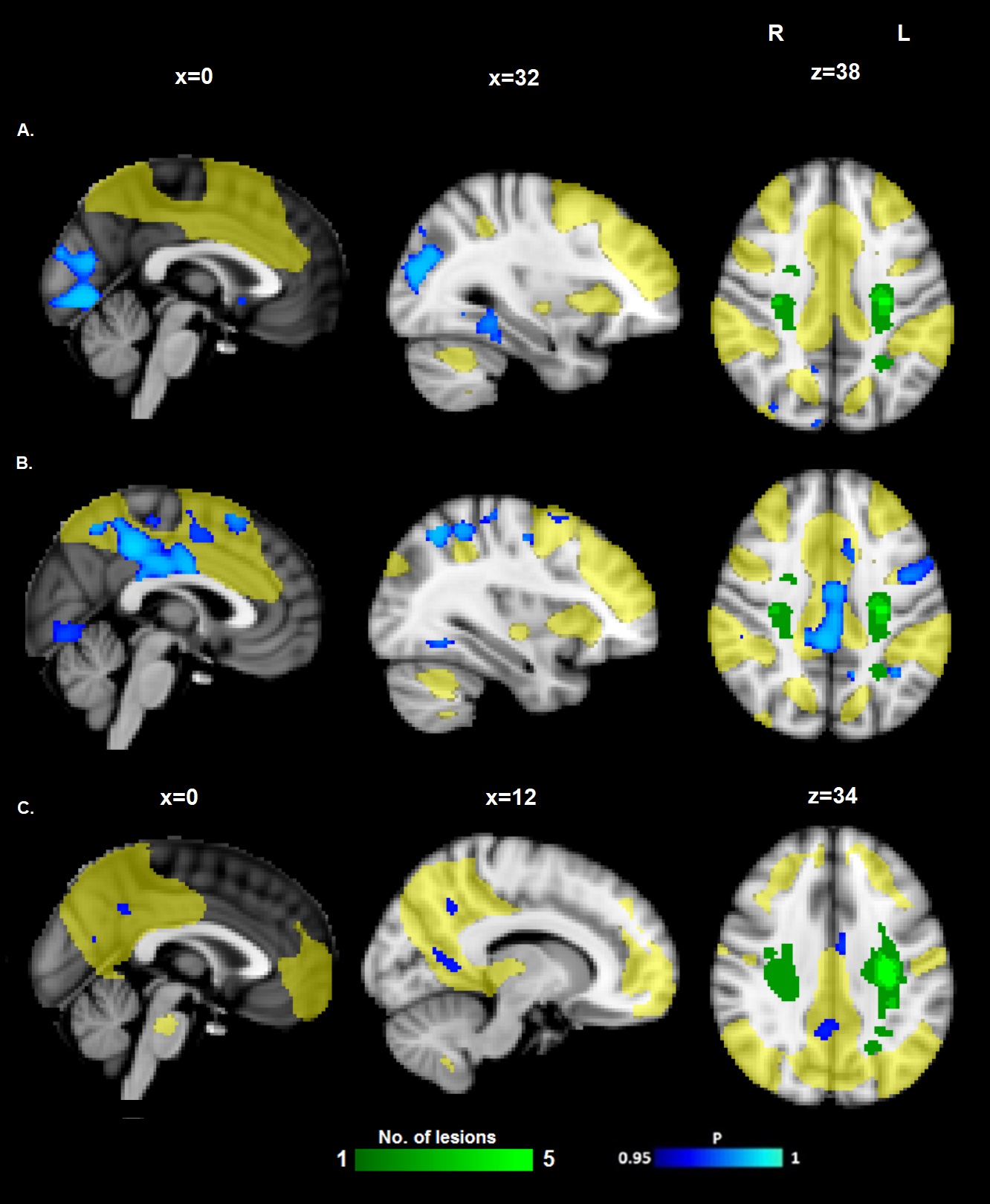


**Figure S4** Resting-state network (RSN) changes related to deficits in cognitive domains in patients with symptomatic carotid artery disease. (A) Difference map (blue-light blue) of CEN where patients with executive dysfunction had decreased functional connectivity compared with patients with normal executive function. (B) Difference map (blue-light blue) of CEN and (C) default mode network (bottom) where patients with abnormal memory had decreased functional connectivity compared with patients with normal memory. The blue-light blue color bar showed the range of family-wise error (FWE)-corrected P value. The green-light green color bar showed the variation between the minimum and maximum number of acute lesions on DTI b0 images. All tests were corrected for age and mean relative displacement.
